# How to Individualize Coronary Assessment and Revascularization in Severe AS Patients Undergoing TAVI in the Era of Lifetime Management?

**DOI:** 10.3390/jcm15103671

**Published:** 2026-05-10

**Authors:** Krzysztof Sobczyk, Miłosz Dziarmaga, Mateusz Dziarmaga, Marek Grygier, Marek Jemielity, Andrzej Wykrętowicz, Anna Olasińska-Wiśniewska

**Affiliations:** 1 Department of Cardiology Intensive Therapy and Internal Medicine, University Clinical Hospital, Przybyszewskiego Street 49, 60-355 Poznan, Poland; 2 Department of Cardiology Intensive Therapy and Internal Medicine, Poznan University of Medical Sciences, Przybyszewskiego Street 49, 60-355 Poznan, Poland; 3 The First Department of Cardiology, Poznan University of Medical Sciences, Długa Street ½, 61-848 Poznan, Poland; marek.grygier@usk.poznan.pl; 4 Department of Cardiac Surgery and Transplantology, Poznan University of Medical Sciences, Długa Street ½, 61-848 Poznan, Poland

**Keywords:** TAVI, coronary revascularization, aortic stenosis, coronary artery disease, coronary computed tomography angiography

## Abstract

Coronary artery disease (CAD) often coexists with severe aortic stenosis (AS) in patients undergoing transcatheter aortic valve implantation (TAVI), posing a complex diagnostic and therapeutic challenge. As TAVI is increasingly used for younger, lower-risk patients, managing CAD is becoming a personalized, long-term clinical concern. This narrative review summarizes the current evidence on coronary assessment and revascularization strategies in individuals with severe AS. Invasive coronary angiography remains the leading method for anatomical coronary imaging, but coronary computed tomography angiography is emerging as a reliable alternative that may reduce unnecessary invasive procedures in certain patients. The routine performance of PCI before TAVI is under increasing scrutiny, and available data support a more selective approach based on lesion significance, CAD complexity, procedural timing, and anticipated need for future coronary access. Significant uncertainties remain concerning the physiological evaluation of lesions, the timing and completeness of revascularization, and the treatment of left main or multivessel disease. Additional phenotype-specific and longitudinal studies are needed to improve management algorithms for this population.

## 1. Introduction

The coexistence of aortic stenosis (AS) and coronary artery disease (CAD) hinders both diagnosis and management and is associated with worse prognosis and higher risk for adverse cardiovascular events. The 2020 ACC/AHA guidelines recommend surgical aortic valve replacement (SAVR) for patients under 65 years or with a life expectancy > 20 years, and transcatheter aortic valve implantation (TAVI) for those over 80 years or with a life expectancy < 10 years. For individuals aged 65–80 years, both approaches are Class I options, with the final decision based on anatomy, comorbidities, and patient preference [1]. This trend is further supported by the updated 2025 ESC/EACTS guidelines for managing valvular heart disease (VHD), which have lowered the age threshold for TAVI from 75 to 70 years in patients with suitable anatomy, transfemoral access feasible, and a tricuspid valve [2]. More recent randomized clinical trials (RCTs) with long-term follow-up, 6- to 7-year, also reinforce this trend. Notable studies include 7-year data from PARTNER 3 and 6-year results from the Evolut Low Risk trial [3,4]. Collectively, these studies indicate that, among patients in their 70s, TAVI achieves outcomes comparable to surgery at midterm follow-up, including mortality, stroke, and rehospitalization [3,4,5]. Prosthesis durability is the main consideration in decision-making regarding TAVI implementation in younger populations. The coexistence of CAD is in this context even more challenging. Optimal management remains demanding due to overlapping symptoms, such as exertional dyspnea and angina, and the interplay of pathophysiological mechanisms. Additionally, AS exacerbates myocardial ischemia and alters coronary hemodynamics, which may confound fractional flow reserve (FFR) measurements [6,7]. This narrative review aims to provide a critical synthesis of current evidence, with particular emphasis on diagnostic strategies and contemporary treatment approaches. Because the definitions of major adverse cardiovascular events (MACE) and major adverse cardiac and cerebrovascular events (MACCE) differed across the studies cited in this paper, these composite endpoints were interpreted according to the original reports. MACE typically included death, myocardial infarction (MI), and occasionally stroke or repeat revascularization, whereas MACCE explicitly included cerebrovascular events.

## 2. Literature Search Strategy and Review Framework

This article is a narrative review that provides a clinically oriented synthesis of the current evidence on the coexistence of CAD and severe AS in patients referred for TAVI. A targeted, non-systematic search of MEDLINE, Embase, and the Cochrane Library was performed to identify relevant publications. Priority was given to clinical practice guidelines, randomized and observational studies, meta-analyses, and key consensus documents addressing epidemiology, diagnostic assessment, prognostic implications, revascularization strategies, procedural timing, and post-TAVI coronary access. Additional references were identified through manual screening of the bibliographies of selected articles. The final selection of studies was based on their clinical importance, methodological quality, and contribution to the interpretation of unresolved or controversial issues. The following search terms were used: “coronary artery disease” OR “CAD” OR “coronary heart disease” OR “CHD” OR “ischemic heart disease” OR “percutaneous coronary intervention” OR “PCI” AND “aortic stenosis” OR “calcific aortic stenosis” OR “AS” AND “transcatheter aortic valve implantation” OR “transcatheter aortic valve replacement” OR “TAVI” OR “TAVR”.

## 3. Epidemiological and Pathophysiological Overlap

### 3.1. Epidemiology and Pathophysiology

The coexistence of AS and CAD reflects active multidirectional mechanisms. They include endothelial injury, genetic factors, lipid accumulation, calcific remodeling, oxidation, and fibroblastic transformation of activated valve interstitial cells [8,9,10,11,12]. Their overlap is linked to an increased inflammatory response and disturbances in trace element balance [13]. The common cardiovascular profile identifies similar risk factors such as diabetes, arterial hypertension, smoking, obesity, and dyslipidemia. There are also differences between AS and CAD, particularly in their remodeling patterns: AS progresses continuously, whereas CAD may undergo abrupt changes, marked by plaque rupture and progression. Available evidence suggests that the prevalence of CAD among patients with severe AS ranges from 38% to 75%, depending on the study population and data source [14,15,16]. Notably, it is present in approximately 45–70% of individuals referred for TAVI [17,18], with absolute numbers increasing following expansion of TAVI indications to low surgical risk populations [19]. An increasing proportion of patients undergoing TAVI are now being pre-procedurally identified with CAD, both in invasive and noninvasive angiography [20]. The clinical relevance of CAD in this population remains incompletely defined, and the reciprocal interactions between AS and CAD remain incompletely understood. Many studies rely on angiographic definitions of CAD, which fail to account for functional significance or disease complexity [6,21]. Latest evidence suggests that the prognostic impact of CAD depends more on its extent and anatomical complexity than on its presence alone [22,23]. Throughout this review, CAD complexity is interpreted in accordance with the definitions used in the cited studies, which variably defined “complex CAD” using mostly either SYNTAX score > 22, or anatomical descriptors such as left main (LM) disease and multivessel disease (MVD).

### 3.2. Clinical Presentation

Symptoms such as angina and dyspnea are common to both CAD and AS, limiting the ability to distinguish these conditions on clinical grounds alone [6]. Angina occurs in up to 40% of patients with severe AS [24], potentially as a consequence of impaired coronary flow reserve (CFR) [25]. Multivariable regression analysis from the SZEGED Study identified lower CFR as an independent predictor of increased cardiovascular morbidity and mortality in patients with AS [26]. Increased left ventricular afterload and elevated end-diastolic pressure contribute to left ventricular hypertrophy, which in turn shortens diastolic coronary filling time (due to prolonged systole), increases extravascular compression of the coronary vessels, and raises oxygen demand. Simultaneously, these changes reduce coronary perfusion pressure, impair microvascular circulation and myocardial blood distribution, and reverse the endocardial/epicardial myocardial blood flow ratio [24,25,27]. These alterations may ultimately lead to subendocardial ischemia and myocardial fibrosis, even without obstructive CAD [25,27,28]. Briefly, as valvular stenosis progresses, elevated systolic load increases myocardial oxygen demand, whereas higher left ventricular filling pressures diminish diastolic coronary supply. The resulting supply–consumption mismatch affects the subendocardium, contributing to ischemia despite angiographically normal epicardial arteries [28]. Although dyspnea is more common in severe AS, it is also frequently present in moderate disease and symptom burden. Therefore, it cannot reliably differentiate moderate from severe disease [29]. In addition, angina and dyspnea may at times be more closely related to concomitant CAD and conduction disturbances than to AS itself [29].

## 4. Diagnostic Challenges in Patients with CAD and Severe AS

### 4.1. Invasive Coronary Angiography

Invasive coronary angiography (ICA) has traditionally been considered the reference standard for anatomical coronary imaging. Patients presenting with proximal stenosis > 70%, LM stenosis > 50%, non-evaluable proximal segments on coronary computed tomography angiography (CCTA), pre-existing cardiovascular disease, severe secondary ventricular mitral regurgitation, left ventricular systolic dysfunction, or symptoms suggestive of myocardial ischemia should be initially referred for ICA [2,6,23,30,31,32]. The 2025 ESC/EACTS Guidelines further extend these indications to include men aged ≥40 years, postmenopausal women, and individuals with one or more cardiovascular risk factors [2]. A major advantage of ICA is the ability to perform ad hoc percutaneous coronary intervention (PCI) in lesions deemed suitable for revascularization. Among the main drawbacks of ICA are its invasive nature, which carries the risk of vascular complications, and the limited reliability of borderline lesion assessment in the absence of adjunctive physiological evaluation [6,33]. In the setting of AS-related alterations in coronary physiology, visual interpretation alone may misclassify stenosis severity, potentially resulting in either unnecessary revascularization or inappropriate deferral of treatment.

### 4.2. Non-Invasive Imaging

According to current guidelines, computed tomography (CT) is a valuable tool for assessing CAD in patients with VHD, particularly during pre-procedural planning for TAVI [2]. Beyond structural evaluation of the aortic valve complex and vascular scanning for feasible transfemoral access, CCTA enables characterization of the coronary arteries [20,30]. CCTA is a non-invasive alternative to ICA that may serve as a triage tool preceding angiography in preoperative assessment, and as a gatekeeper strategy to reduce unnecessary invasive testing [34]. In recent years, evidence supporting the use of CCTA over ICA in this clinical indication has increased [20,30,31,35,36]. In 2025, the ESC/EACTS guidelines for the management of VHD updated CCTA to Class I, Level of Evidence B, and recommended it before valve intervention in patients with moderate or lower (50%) pretest likelihood of CAD [2]. Pre-TAVI planning using CCTA appears feasible and safe, achieving procedural and clinical outcomes comparable to those of ICA [30,32]. Gatti et al. reported a sensitivity of 97% and a specificity of 68% for CCTA in detecting obstructive CAD in patients referred for TAVI [37]. This approach allows for a 40.9% reduction in unnecessary ICA procedures [37]. Principal limitations of CCTA include common suboptimal pharmacological preparation, tachyarrhythmias, including atrial fibrillation, and elevated coronary artery calcium burden. The latter leads to blooming artifacts and increased false-positive findings, which reduce diagnostic specificity [37]. Historically, concerns about hemodynamic instability in patients with severe AS have limited the use of pharmacological optimization protocols, as nitroglycerin and beta-blockers have been traditionally avoided [38]. However, growing evidence suggests these agents can be used safely, with transient hypotension reported in approximately 1.2% of cases [30,35]. Nowadays, CCTA with adjunctive nitroglycerin and selective beta-blockers or ivabradine use appears effective for CAD evaluation in stable severe AS [30,35,38,39]. The Agatston score, which remains the clinical reference standard for assessing coronary artery calcification, may also be used in patients referred for TAVI to support decision-making regarding ICA/PCI [40,41]. Because it is derived from a non-contrast, ECG-gated CT scan, it avoids the need for medical optimization and is relatively insensitive to calcium blooming [41,42]. In this setting, Agatston thresholds of >170 for the entire coronary tree, >117 for the proximal segments, >22 for the proximal right coronary artery (RCA), and >30 for the proximal left anterior descending (LAD) and left circumflex (LCx) provided 90% sensitivity for significant CAD [41]. When a sensitivity target of at least 98% was applied, the corresponding thresholds were >2 for the RCA, >3 for the LAD, and >27 for the LCx [41]. Advances are also present in image acquisition and post-processing. Third-generation dual-source CT with an ECG-triggered ultra-high-pitch protocol has been shown to improve image quality and diagnostic performance while reducing radiation and contrast exposure [43,44]. Its rapid data acquisition may also help to mitigate motion-related limitations in patients with high or irregular heart rhythms [44]. Photon-counting detector CT, which is currently gaining interest, offers spectral imaging with improved tissue contrast, higher spatial resolution, and reduced partial-volume effects, potentially limiting calcium blooming artifacts [45,46,47]. In a study by Hussain et al., Artificial Intelligence–Quantified Coronary Plaque Analysis (AI-QCPA) applied to pre-TAVI CCTA enabled quantitative assessment of coronary plaque burden and showed that greater total, calcified, non-calcified, and low-attenuation plaque volumes were associated with an increased risk of MACE [48]. In contrast, Fang et al. found no significant association between high-risk plaque characteristics (HRPC), such as positive remodeling and low-attenuation plaque, and MACE [49]. Moreover, none of the individual HRPC components was significantly associated with adverse outcomes. However, these studies may differ in methodological details, including scanning protocols and reconstruction algorithms, so their results should be interpreted carefully. Quantitative computed tomography angiography (CTA) analysis of aortic valve tissue volume and composition has been shown to assist in the diagnosis of severe AS and to help differentiate between high-gradient and low-flow, low-gradient AS [50]. According to Grodecki et al., this method may also be useful for predicting 30-day MACE after TAVI [50]. Findings reported by Lecomte et al. suggest that coronary artery evaluation on pre-TAVI CCTA with deep-learning reconstruction and motion-correction algorithms may reduce the need for ICA, potentially allowing its safe omission in 47% of patients [51]. Pre-procedural CTA using machine-learning models integrating imaging with clinical data also provided a more accurate prediction of 1-year all-cause mortality after TAVI than clinical assessment or EuroScore II alone [52].

### 4.3. Cardiac Magnetic Resonance

Although echocardiography and CT remain the primary imaging modalities for assessing AS severity, anatomical characteristics, and procedural planning, cardiac magnetic resonance (CMR) still plays a supportive role in the comprehensive evaluation of these patients. Mantini et al. conducted a head-to-head comparison of CMR and echocardiography (including both transesophageal (TEE) and transthoracic (TTE)) for grading AS. CMR-derived aortic valve area (AVA) by planimetry showed a strong correlation with TEE (CCC = 0.85; increasing to 0.93 after excluding patients with heavily calcified valves), while effective orifice area (EOA) correlated well with TTE measurements (CCC = 0.82) [53]. Moreover, Thornton et al. demonstrated the utility of CMR in patients with low-flow AS, showing that CMR-derived stroke volume index enables accurate phenotyping of AS flow states and provides independent prognostic information, with lower values associated with increased mortality after valve intervention [54]. According to a prospective, randomized trial by Reindl et al., CMR is not inferior to CT for procedural planning of transcatheter aortic valve interventions, providing comparable assessment of aortic root anatomy, annular dimensions, and vascular access [55]. Although CT may overestimate and CMR may underestimate calcification, these differences do not appear to affect valve sizing, access route selection, or procedural outcomes. Stress CMR has an established role in the evaluation of patients with coronary artery disease, providing high diagnostic and prognostic value [56]. Importantly, although traditionally considered contraindicated in aortic stenosis, Salatzki et al. showed that stress CMR can be performed safely and effectively even in patients with moderate to severe AS [57]. In summary, although not a first-line imaging modality, CMR is a valuable complementary tool in aortic stenosis and coronary artery disease, providing additional diagnostic, anatomical, and prognostic information.

### 4.4. Physiological Assessment

ICA may not accurately reflect the significance of (especially intermediate) lesions in the setting of altered coronary physiology driven by severe AS. Aortic valve replacement also influences coronary hemodynamics, often improving coronary flow reserve and potentially modifying the functional significance of coronary stenoses, including unmasking significant ischemia by improving microvascular vasodilatory capacity [58]. This is clinically relevant because accurate physiological evaluation often guides revascularization decisions. Ribichini et al. found that FFR-guided PCI was associated with a significantly lower incidence of major adverse cardiac and cerebrovascular events (MACCE) than angiography-guided PCI at 1-year follow-up [59]. The functional severity of CAD may be underestimated in FFR measurements since invasive FFR values may decrease after TAVI [60,61]. Non-hyperemic pressure ratios, such as instantaneous wave-free ratio (iFR), likely overestimate functional severity instead [60,62]. However, the available evidence is not consistent. For instance, according to the study by Ahmad et al., flow during the wave-free period of diastole remains stable over the TAVI procedure, as do iFR values [61]. In contrast, Scarsini et al. assessed iFR before and after TAVI across coronary lesions of varying angiographic severity. In the entire study population, mean iFR values before and after TAVI were similar [63]. At the level of individual lesions, unpredictable iFR fluctuations were observed that exceeded the threshold of functional significance, either favorably or unfavorably [63]. The magnitude of these changes was probably reflected in the reduction in the transaortic gradient, which was greater as the gradient decreased after treatment [63]. This could change the indications for revascularization between the pre- and post-TAVI assessments and highlights that iFR should be interpreted with caution in patients with severe AS undergoing TAVI. Otherwise, it may inadequately reflect the hemodynamic significance of a given lesion. In line with these findings, Jo et al. reported that FFR is less dependent on hemodynamic disturbances induced by severe AS and provides a stronger prognostic significance [62]. Nonetheless, iFR may still be considered an eligible rule-out tool for hemodynamically significant lesions, as it showed high sensitivity and a high negative predictive value at the 0.89 threshold for predicting FFR ≤0.80 [62,63]. CT fractional flow reserve (CT-FFR) is a noninvasive method for quantifying the hemodynamic significance of coronary stenoses. Compared with anatomical CCTA assessment alone, CT-FFR demonstrated improved diagnostic accuracy [23,35]. Its independence from labile hemodynamic factors may confer particular diagnostic value while avoiding unnecessary ICA [61]. CT-FFR ≤ 0.80 independently predicted an increased risk of MACE, and its prognostic value was further enhanced when integrated with the Optimal Antiplatelet Therapy for Chinese Patients with Coronary Artery Disease (OPT-CAD) score [49]. Wang et al. assessed the utility of machine learning-based CT-FFR derived from CCTA in the diagnosis of concomitant CAD [23]. CT-FFR outperformed CCTA in most diagnostic metrics; however, it showed lower specificity and a higher false-positive rate [23]. Overall, the study suggested that machine learning-based CT-FFR effectively targets coronary hemodynamic abnormalities and, when integrated with preoperative CCTA, may serve as a useful non-invasive approach for ruling out significant CAD in patients evaluated for TAVI [23]. Overall, FFR and iFR provide complementary rather than equivalent information. FFR may offer stronger prognostic value but can be influenced by hemodynamic alterations related to AS, whereas iFR is less dependent on hyperemic flow and may be useful as a rule-out tool, though values close to the diagnostic threshold warrant caution.

## 5. Heart Team Decision-Making and Future Coronary Access

Individualized decision-making within a multidisciplinary Heart Team framework is essential and should integrate anatomical, functional, and clinical variables [7,64]. Prosthesis design and implantation-related factors, particularly frame height, cell design, leaflet position, implantation depth, and commissural alignment, influence coronary access after TAVI [64]. In particular, the transcatheter heart valve (THV) leaflet height relative to annular implantation depth appears to be a more important determinant of post-TAVI coronary access than commissural post height alone [64]. Accordingly, future coronary access after TAVI is largely influenced by THV design. In the CAvEAT study by Tarantini et al., the short-frame SAPIEN 3/Ultra prosthesis was associated with the highest rate of selective coronary access following TAVI [65]. Among tall-frame valves, Portico/Navitor and ACURATE neo/neo2 were associated with more favorable coronary access than the closed-cell Evolut Pro/Pro+ system [65]. Although future coronary access should not be the sole factor guiding THV selection, it remains an important consideration in lifetime management, particularly in younger patients and in those with a higher likelihood of requiring subsequent coronary intervention or redo-TAVI [64,66].

### Coronary Obstruction

Coronary artery obstruction is a rare (0.6–0.8%) but serious complication of TAVI, with a mortality rate exceeding 50% [67]. It occurs when the leaflets of the native valve or a surgical prosthesis are displaced by the transcatheter prosthesis, thereby blocking the coronary ostia. The valve-in-valve TAVI is a particularly challenging procedure, burdened with a 4 to 6 times higher risk of coronary artery obstruction [68,69], and is highest for stentless or stented bioprosthesis with externally mounted leaflets [70]. Rescue treatment requires bailout PCI or emergency coronary artery bypass grafting (CABG). Several preventive techniques have been proposed to prevent this devastating complication. Coronary protection is commonly achieved by placing a “chimney stent” protruding into the aorta beyond the valve, which deflects the leaflet [67]. Leaflet modification technique, called bioprosthetic or native aortic scallop intentional laceration to prevent coronary artery obstruction (BASILICA), was first proposed and introduced by Khan and his team [71] in 2018. It was derived from the LAMPOON (intentional laceration of the anterior mitral leaflet to prevent left ventricular outflow obstruction) mitral valve procedure. Pre-procedurally, CT is required to assess the risk of obstruction and plan BASILICA [72]. BASILICA is performed by transcatheter electrosurgical leaflet splitting, preceding transcatheter prosthetic implantation, by slicing the native or bioprosthetic leaflet in front of the coronary ostium to preserve coronary flow after TAVI. Early [73] and multicentre registries [74] showed a high procedural success rate (88%) and low 30-day mortality rate (less than 3%). Further technique modifications include balloon-assisted BASILICA (BA-BASILICA), developed to overcome the risk of inadequate splay [75]. After leaflet traversal, the traversal point is dilated with a non-compliant balloon or a lithotripsy balloon. Potential complications of the BASILICA technique include vascular access-site complications, periprocedural stroke, and injury of non-target structures, and result from technical challenges related to the need for catheter exchange and extensive manipulation of highly degenerated leaflets [76]. The BASILICA technique can be supported by TEE and intravascular ultrasound imaging to precisely position the devices at the base of the target leaflet, assess the sinus of Valsalva with coronary ostia, and facilitate early detection of adverse events [77,78,79]. Other new techniques include mechanical leaflet splitting with ShortCut (Pi-Cardia), TELLTALE device, or the UNICORN technique [67], or the CATHEDRAL technique; however, the experience with their use is still limited.

## 6. Prognostic Significance of CAD

The prognostic relevance of CAD in patients undergoing TAVI remains uncertain, as many studies have yielded conflicting results. Although CAD is a well-established predictor of increased mortality in the general population, several studies suggest that its presence alone may not adversely affect outcomes after TAVI [80,81,82,83]. CAD has not been shown to affect either short- or long-term survival. While evidence is limited regarding LM disease, there is some suggestion that significant LAD stenosis may have a detrimental effect [80]. Although chronic total occlusions (CTOs) have been associated with a higher risk of in-hospital events, no corresponding association with increased mortality has been demonstrated [81,84]. Increased mortality after TAVI was described as evident only in patients with severe CAD and was not observed in individuals with a lower CAD burden [83]. Some observational studies with contrasting outcomes deserve more attention. Stefanini et al. reported an association between greater CAD severity and unfavorable 1-year clinical outcomes after TAVI [85]. In their study, patients with a preoperative SYNTAX score > 22 had a higher risk of MACE, possibly reflecting less complete revascularization than in patients without CAD or with a SYNTAX score < 22 [85]. Conversely, Paradis et al. reported no significant association between CAD presence or incomplete revascularization and outcomes at 30 days and 1 year following TAVI [86]. These studies also present certain limitations. They were conducted about a decade ago, included relatively small study populations, and may also have been influenced by methodological differences. According to Huczek et al., the coexistence of obstructive CAD in patients referred for TAVI is an independent negative prognostic factor for early mortality, but revascularization before valve replacement appears to improve survival rates [87]. In the East Denmark Heart Registry, no significant differences in long-term survival were observed among patients without obstructive CAD, patients with obstructive CAD who did not undergo pre-TAVI revascularization, and those with obstructive CAD who underwent pre-TAVI revascularization [84]. The incidence of post-TAVI revascularization did not differ significantly between these groups [84]. Survival was also not affected by prior CABG [84]. Similar findings were reported in the FRANCE-2 registry, in which neither the presence nor the distribution of CAD was associated with higher 3-year mortality after TAVI in the high-risk population, considered unsuitable for SAVR [80]. The prognostic value of the SYNTAX score in patients with concomitant severe AS has also been questioned by some authors [87], whereas others have reported that higher SYNTAX scores, reflecting greater CAD complexity, are associated with a worse prognosis [85]. The presence of CAD alone probably does not worsen outcomes after TAVI, whereas disease complexity appears to carry greater prognostic relevance [23]. However, determining which patients truly benefit from revascularization remains challenging, particularly in the absence of RCTs.

## 7. Revascularization Strategies

Although the combination of ICA and PCI is commonly regarded as sufficient for evaluating and treating most proximal coronary lesions, with ≥70% stenosis in non-LM vessels and ≥50% in the LM typically deemed significant, the precise threshold that differentiates intermediate lesions requiring revascularization from those suitable for conservative management remains uncertain. Comparative evidence on integrated treatment approaches is available (Table 1). In recent studies by Taghiyev et al. and Stundl et al., outcomes after TAVI + PCI were generally comparable to those after SAVR + CABG across early, midterm, and long-term follow-up [88,89]. Importantly, these analyses included patients with anatomically complex CAD, defined as significant untreated LM stenosis (>50%) or MVD in the context of severe AS burden [88]. Patients treated with SAVR + CABG, although more frequently affected by early bleeding and stroke, did not have higher early mortality and showed a slightly lower 3- and 5-year mortality rate than those undergoing TAVI + PCI [89,90]. Consistent with the outlined findings, Alperi et al. observed similar 3-year MACCE rates for TAVI + PCI and SAVR + CABG in patients with complex CAD (defined as significant >50% unprotected LM CAD or the presence of an anatomical SYNTAX score > 22), but with a higher risk of repeat revascularization in the TAVI + PCI group [91]. In a subgroup analysis of the SURTAVI trial, TAVI + PCI was shown to be a reasonable alternative to SAVR + CABG in intermediate-risk patients with severe AS and non-complex CAD, with outcomes similar to those of SAVR + CABG at 2 years [92,93]. Recent meta-analyses have provided new time-dependent comparisons between surgical and transcatheter approaches. One such analysis, including 53,869 patients, showed that TAVI + PCI was associated with more favorable 30-day outcomes, including fewer strokes, MIs, and acute kidney injuries (AKIs), but with a higher rate of pacemaker implantation [94]. This early safety profile contrasts with mid- to long-term observations, in which TAVI + PCI was associated with higher risks of all-cause mortality, MI, and repeat revascularization [94]. The stroke outcomes remained broadly unchanged during follow-up [94]. Importantly, the certainty of evidence was limited [94]. A Kaplan–Meier-derived meta-analysis by Baudo et al., including 1998 patients, presented similar findings: an early benefit of TAVI + PCI in patients with severe AS, but followed by a trend reversal after approximately 73 days in favor of SAVR + CABG [95]. An earlier meta-analysis including 104,220 patients with a mean follow-up of 30.2 months reported higher rates of all-cause mortality, repeat coronary reintervention, and periprocedural vascular complications in patients undergoing TAVI + PCI [96]. Many studies suggest that EuroSCORE II and STS PROM scores provide a practical framework for mortality risk estimation [88,89,97].

### 7.1. Surgical Approach

SAVR combined with CABG offers complete revascularization and remains a preferred strategy in patients with complex coronary anatomy and acceptable surgical risk [94,103]. The 2025 ESC/EACTS Guidelines for the management of VHD recommend CABG as a suitable option for patients undergoing aortic valve replacement who have ≥70% stenosis of any major epicardial coronary vessel or ≥50% stenosis of the LM [2]. A similar approach is reflected by the 2020 ACC/AHA guidelines. For patients with intermediate CAD (stenosis 40–69%), CABG should be considered [2]. Guidelines also note that physiological tests, such as FFR, are not yet validated for decision-making in the setting of concomitant VHD [2]. The advantage of the SAVR + CABG strategy is the availability of solid long-term outcome data [104]. This is supported by retrospective analyses of surgically treated patients from the “pre-TAVI era”, which reported 10-year survival of 42.6%, cardiac mortality of 7.2%, and MACCE-free survival of 55.1% in elderly, intermediate-risk individuals [104]. Because patients undergoing TAVI + PCI often differ substantially in baseline clinical profile, broad head-to-head comparisons with similarly long follow-up remain difficult to obtain. As indications for TAVI + PCI continue to expand toward younger and intermediate-risk patients, future studies may provide more informative long-term comparative data.

### 7.2. Transcatheter Approach

A meta-analysis of observational studies showed that TAVI + PCI is a reasonable alternative to SAVR + CABG, highlighting its positive impact on long-term mortality, lower risk of atrial fibrillation, shorter hospitalization, and favorable outcomes in the elderly population and in patients with kidney disease [105]. According to Wilimski et al., a history of revascularization (PCI or CABG) before TAVI was not associated with 30-day or 12-month all-cause mortality, although higher post-TAVI stroke rates were observed in previously revascularized patients [97]. Thus, the authors suggested that prior revascularization should be considered a marker of a higher-risk clinical profile rather than an independent mortality determinant, and they support the feasibility of TAVI for the treatment of severe AS in individuals with a history of revascularization [97]. The randomized NOTION-3 trial provided a different perspective by comparing staged PCI + TAVI, in selected cases, with conservative management + TAVI [102]. At a median follow-up of 2 years, the PCI-based approach (performed in individuals with FFR ≤ 0.80 or an angiographic coronary stenosis ≥ 90%) demonstrated a 10 percentage-point lower risk of the primary endpoint, which was defined as a composite of death from any cause, MI, or urgent revascularization [102]. The 2025 ESC/EACTS Guidelines for the management of VHD favor TAVI in anatomical findings such as porcelain aorta, severe chest deformation, or intact post-CABG grafts [2]. Concerns persist regarding incomplete revascularization, lesion selection, and optimal timing [7,21,33,103,105].

### 7.3. Incomplete Revascularization

The evidence regarding incomplete revascularization in patients with CAD undergoing TAVI remains conflicting, and consensus is lacking. Studies demonstrate variable outcomes depending on the definition of incomplete revascularization, duration of follow-up, and severity of CAD. While some publications, such as the NOTION-3 [102] study, seem to support complete revascularization, others, such as the REVASC-TAVI registry [101], do not show that complete revascularization is associated with a significant difference in MACE risk compared to incomplete revascularization. To objectively quantify residual coronary disease after revascularization, the residual SYNTAX score has been introduced. Similar to the baseline SYNTAX score, attempts have been made to evaluate the prognostic utility of residual SYNTAX in patients undergoing TAVI and to identify clinically relevant cut-off values [83,85,106]. In this context, a residual SYNTAX > 8 (according to some authors, >14 [85]) was used to define incomplete revascularization and was associated with increased mortality rates post-TAVI, stroke, or MI [83,85]. Conversely, a post-revascularization residual SYNTAX score < 8 appears to identify a lower residual CAD burden and has been described by Witberg et al. as “reasonable” incomplete revascularization [83]. In our view, current evidence and recommendations are often interpreted broadly across various CAD phenotypes in patients undergoing TAVI (Table 1). Non-complex CAD, MVD, and LM involvement should rather be considered distinct clinical and anatomical scenarios. A limitation of the available evidence is its non-randomized nature, together with inconsistent characterization of CAD extent. In several studies, complex CAD is considered as a binary variable, typically defined by a SYNTAX score > 22, without separately distinguishing between LM involvement or MVD. Consequently, patients are often grouped within this broad category. In other studies, these specific disease variants are often excluded. However, the TAVR-LM registry provides an interesting exception, as it evaluated outcomes focusing on patients undergoing TAVI with LM PCI [98]. This publication offers a broad comparative analysis, including TAVI + LM PCI versus TAVI alone, protected versus unprotected LM PCI, different timing of LM PCI in relation to TAVI, and the impact of pre-existing LM stents [98]. In matched analyses, 1-year mortality after TAVI + LM PCI was comparable to TAVI alone, and outcomes were not significantly influenced by LM protection status, PCI timing, or stent landing zone [98]. In the same study, patients undergoing bailout LM PCI in TAVI-related coronary complications had higher early mortality and a numerically higher 1-year mortality than those with planned LM PCI for pre-existing LM obstruction [98]. Future research should move beyond a simplified no-CAD/non-complex CAD/complex CAD framework based mainly on the SYNTAX score and adopt phenotype-dependent stratification, distinguishing limited CAD from MVD with or without LM involvement. This approach can enhance future outcome predictions, promote personalized treatment strategies, and assist in selecting THV. In this context, LM stenosis and MVD should not be solely considered as elements of a higher SYNTAX score.

### 7.4. Strategy Selection

The choice of revascularization strategy should extend beyond a simple PCI-versus-CABG framework and be individualized, based on coronary anatomical complexity, surgical risk, life expectancy, and the anticipated feasibility of future coronary access [7,64]. The TCW study tested the non-inferiority of FFR-guided TAVI + PCI vs. SAVR + CABG in patients with severe AS and complex CAD [99]. Although the results suggested a potential benefit for clinical outcomes with an FFR-guided revascularization strategy, these findings require confirmation in larger RCTs with longer follow-up [99]. In summary, no single approach has demonstrated universal superiority.

## 8. Timing of PCI Relative to TAVI

There is an ongoing debate concerning the optimal timing of PCI, as published studies have yielded conflicting results. No clear consensus has yet been reached on whether revascularization should be performed before, after, or even simultaneously with THV implantation. The most common framework, Pre-TAVI, i.e., staged PCI, may theoretically prevent procedural ischemia and facilitate coronary access [33]. A disadvantage of this approach is probably a higher risk of bleeding events and of the composite clinical endpoint of all-cause mortality, stroke, MI, and hospitalization for heart failure (HF) at 2-year follow-up [33,107], although not all available evidence uniformly supports this finding [93,102,108]. This issue is of clinical importance, as acute coronary syndromes occur in less than 5% of patients following TAVI, but STEMI in particular carries mortality up to ~30% at 30 days [33]. Yet the data are largely observational and require caution; for PCI performed concomitantly with TAVI, outcomes are rather unfavorable. It generally increases procedure complexity, contrast exposure (resulting in a higher risk of AKI), early-to-late mortality, and the incidence of the composite endpoint [33,105,107,109]. On the other hand, simultaneous PCI and TAVI may reduce bleeding risk [110], procedural burden, and costs through single arterial access, but these advantages still do not seem to outweigh the cons [33,93,111]. It is considered an uncommon approach, but it should not be completely ruled out, at least in stable, appropriately selected patients [93,110]. In light of the most recent evidence suggesting a survival benefit with a single-session approach, further studies are still required [110]. The lowest 2-year mortality and the lowest composite endpoint rate were observed in PCI after the TAVI strategy [33,107], possibly reflecting a more reliable physiological assessment of CAD. The safety and efficacy of an initially conservative strategy, with PCI performed after TAVI, is under debate. Recent randomized evidence from the PRO-TAVI trial suggests that this approach may be at least non-inferior to PCI before TAVI [112]. However, in the REVASC-TAVI REGISTRY [107], it was associated with a higher incidence of MI and HF rehospitalization. It should be acknowledged that PCI, especially a multistage approach in patients with MVD, may delay TAVI. Each of the presented strategies has its advantages and disadvantages, and the selection of the most appropriate one should be individualized and patient-centered [2,93,112]. In summary, the management of CAD in AS patients remains a topic of long-standing debate, particularly with the improvements in TAVI techniques and operators’ skills. Previous strategies favored coronary revascularization before TAVI to avoid ischemia during the procedure and challenges with coronary access after prosthesis implantation. In contrast, bleeding complications related to dual antiplatelet therapy were a significant concern. Currently, we are witnessing a shift in the perception of coronary artery disease, its significance in patients with aortic stenosis, and the need for early revascularization. The trend to defer PCI until after TAVI is currently gaining more interest. Moreover, concerns regarding prosthesis durability affect the management, particularly making the TAVI strategy more cautious in younger patients. Therefore, lifetime management planning focuses on many factors, as younger patients may require future valve-in-valve implantations, with further challenges in the coronary access with two prostheses. This issue alters the lifetime management paradigm, as the feasibility of a future procedure should inform the choice of the first implanted prosthesis. Ongoing trials and registries may clarify these concerns.

## 9. Limitations of the Current Literature and Future Research Directions

Many questions remain unanswered, limiting the formulation of definitive evidence-based recommendations. Reaching a consensus is challenging because published studies have produced conflicting results, and a substantial proportion of the available evidence comes from non-randomized trials. Additionally, the existing literature is constrained by methodological and clinical limitations. Foremost, the majority of completed RCTs have enrolled patients with relatively low coronary anatomical complexity, limiting the generalizability of findings to patients with MVD/LM disease. Also, patients with recent acute coronary syndrome have been systematically excluded from these trials, precluding extrapolation of results to high-risk subgroups. Secondly, optimal timing of PCI relative to TAVI remains undefined, as existing studies have employed different protocols with variable intervals between procedures. Thirdly, the effectiveness of complete versus incomplete revascularization strategies has not been rigorously evaluated in RCTs. Finally, invasive physiological measurements may be influenced in the setting of severe AS, decreasing diagnostic accuracy and impacting future management. As TAVI indications expand to younger patients, the future is likely to bring more long-term comparative data across all major areas of interest, from diagnostics and treatment to follow-up. As a “hot topic”, current research is particularly focused on the optimal procedural sequencing, whether revascularization should be performed before or after valve implantation. Molecular diagnostics may also represent an emerging area of investigation. Fang et al. presented fibrinogen-like protein 1 (FGL1) as a promising diagnostic and prognostic biomarker [113]. Elevated serum FGL1 in patients with AS-CAD enabled effective differentiation from isolated AS [113]. Unfortunately, its wider use is currently limited by the absence of a standardized clinical detection platform for FGL1 [113]. It is believed that the ongoing investigations will provide essential data to support AI-based algorithms for individualized diagnostics and treatment, patient-centered decision-making, and future guideline recommendations for this complex and growing population.

## 10. Conclusions

In patients with severe AS undergoing TAVI, concomitant CAD should be managed with an individualized, Heart Team-based approach rather than a routine ICA/PCI-first strategy. CCTA becomes an eligible gatekeeper strategy to ICA, and revascularization decisions should consider lesion significance, CAD complexity, and procedural timing. Major unresolved issues include the physiologic assessment of coronary lesions, procedural timing, and the anticipated need for future coronary access. Data on the optimal management of MVD or LM disease are lacking, as prior studies have largely relied on generalized indices of CAD severity. More long-term, phenotype-specific evidence is needed to establish consistent decision-making algorithms.

## Figures and Tables

**Table 1:** Key contemporary and influential studies and their focus on left main, multivessel, and complex coronary artery disease in patients with severe aortic stenosis.

Study	Study Design/Population	LM/MVD/Complex * CAD Relevance	Main Comparison or Focus	Key Findings
Alperi et al., 2021 [91]	Multicenter observational study with propensity score matching; 156 matched patient pairs.	Direct LM/complex CAD evidence. Complex CAD was defined as SS > 22 or unprotected LM disease.	TAVI + PCI vs. SAVR + CABG.	TAVI + PCI and SAVR + CABG had similar MACCE rates at 3-year follow-up, but repeat coronary revascularization was more frequent after TAVI + PCI.
TAVR-LM Registry/Chakravarty et al. (2016) [98]	Multicenter observational registry; 204 high-risk patients undergoing TAVI + LM PCI.	Direct LM evidence. Directly focused on LM stenting, including protected and unprotected LM.	Outcomes of TAVI + LM PCI compared with TAVI controls; planned vs. unplanned LM PCI.	Planned LM PCI in patients undergoing TAVI was associated with outcomes comparable to matched TAVI-only controls in short- and intermediate-term follow-up, whereas bailout LM PCI was associated with worse prognosis.
TCW trial, Kedhi et al., 2024 [99]	Multicenter, open-label randomized trial; 172 patients, aged ≥70 years with severe AS and concomitant complex CAD.	Direct MVD/CAD complexity evidence. Included MVD or advanced CAD; not a pure LM trial. Complex LM anatomy was restricted, with LM bifurcation disease excluded.	FFR-guided PCI + TAVI vs. SAVR + CABG.	FFR-guided PCI + TAVI reduced the 1-year primary endpoint compared with SAVR + CABG: 4% vs. 23%.
SURTAVI CAD substudy/Søndergaard et al., 2019 [92]	Substudy of the randomized SURTAVI trial; 304 intermediate-risk patients with severe AS and noncomplex CAD.	High-impact study not applicable to LM/MVD, but an important comparator for evidence. Complex CAD was excluded; SS > 22 was an exclusion criterion.	Complete percutaneous strategy vs. surgical strategy: TAVI + PCI vs. SAVR + CABG.	TAVI + PCI and SAVR + CABG had comparable 2-year rates of death or disabling stroke in the study population.
Stefanini et al., 2014 [85]	Prospective registry; 445 patients undergoing TAVI.	Relevant for CAD complexity. SS-based disease severity quantification, but without a detailed vascular distinction.	Prognostic impact of CAD severity according to baseline and rSS.	SS > 22 was related to a higher 1-year cardiovascular death, stroke, or MI after TAVI; high rSS also predicted worse outcomes.
Witberg et al., 2017 [83]	Observational cohort: 1270 severe AS patients undergoing TAVI.	Relevant for CAD complexity. Focus on CAD severity and residual coronary disease burden.	Prognostic impact of severe CAD and incomplete revascularization after TAVI.	Severe CAD and incomplete revascularization were associated with increased mortality after TAVI.
Paradis et al., 2017 [86]	Retrospective multicenter cohort; 377 TAVI patients with SS-guided CAD assessment.	Moderately relevant. SS and rSS-based disease severity quantification, but without a detailed vascular distinction.	Clinical relevance of CAD severity and completeness of revascularization after TAVI.	CAD severity and incomplete revascularization were not associated with worse 30-day or 1-year outcomes after TAVI.
FRANCE-2 registry/Puymirat et al., 2017 [80]	National French TAVI registry; 4201 patients.	Moderately relevant. Focus on CAD extent and lesion distribution rather than LM/MVD management.	Prognostic impact of CAD burden in patients undergoing TAVI.	CAD mere presence and vessel count were not associated with higher 3-year mortality, whereas significant LAD disease may be associated with decreased survival rates and greater need for revascularization.
Khawaja et al., 2014 [100]	Observational TAVI cohort: 271 patients treated with Edwards SAPIEN or Edwards SAPIEN XT bioprostheses.	Moderately relevant. Not LM/MVD-specific, but evaluates CAD burden by QCA and SS.	QCA and SS as predictors after TAVI.	SS score > 9 independently predicted mortality, whereas QCA-assessed CAD severity was less informative.
REVASC-TAVI registry/Costa et al., 2022 [101]	Multicenter retrospective registry; 2025 TAVI patients with significant stable CAD (675 propensity score-matched pairs).	Moderately relevant for MVD/revascularization completeness, but not LM-specific.	Complete vs. incomplete revascularization, staged or concomitant with TAVI.	Completeness of revascularization did not impact 2-year mortality or the composite of death, stroke, MI, or HF rehospitalization.
NOTION-3, Lønborg et al., 2024 [102]	Randomized trial; 455 elderly patients with hemodynamically significant CAD undergoing TAVI.	High-impact study, but not applicable to LM/MVD. LM stenosis excluded; median number of significant lesions was 1.	TAVI + PCI vs. TAVI + conservative CAD treatment in patients with significant stable CAD.	PCI reduced the composite of all-cause death, MI, or urgent revascularization: 26% vs. 36%.

* Definitions varied across studies and are explained in the main text. Abbreviations: AS—aortic stenosis; CABG—coronary artery bypass grafting; CAD—coronary artery disease; FFR—fractional flow reserve; HF—heart failure; LAD—left anterior descending artery; LM—left main coronary artery; MACCE—major adverse cardiac and cerebrovascular events; MI—myocardial infarction; MVD—multivessel disease; PCI—percutaneous coronary intervention; QCA—quantitative coronary angiography; SAVR—surgical aortic valve replacement; SS—SYNTAX score; rSS—residual SYNTAX score; TAVI/TAVR—transcatheter aortic valve implantation/replacement.

## Data Availability

No new data were created or analyzed in this study. Data sharing is not applicable to this article.

## Abbreviations

The following abbreviations are used in this manuscript:

AI—artificial intelligence; AS—aortic stenosis; AVA—aortic valve area; CAD—coronary artery disease; CCTA—coronary computed tomography angiography; CT—computed tomography; CTA—computed tomography angiography; EOA—effective orifice area; HF—heart failure; HRPC—high-risk plaque characteristics; ICA—invasive coronary angiography; LAD—left anterior descending artery; LCx—left circumflex artery; LM—left main; MACE—major adverse cardiovascular events; MACCE—major adverse cardiac and cerebrovascular events; MVD—multivessel disease; RCA—right coronary artery; RCT—randomized controlled trial; SAVR—surgical aortic valve replacement; TAVI—transcatheter aortic valve implantation; TEE—transesophageal echocardiography; THV—transcatheter heart valve; TTE—transthoracic echocardiography; VHD—valvular heart disease.
